# Esophageal cancer research today and tomorrow: Lessons from algae and other perspectives

**DOI:** 10.3934/genet.2018.1.75

**Published:** 2018-03-07

**Authors:** Vladlena Tiasto, Valeriia Mikhailova, Valeriia Gulaia, Valeriia Vikhareva, Boris Zorin, Alexandra Kalitnik, Alexander Kagansky

**Affiliations:** 1 Centre for Genomic and Regenerative Medicine, School of Biomedicine, FEFU, 8 Sukhanova str, Vladivostok, Primorsky region, 690950, Russian Federation; 2 Laboratory of Pharmacology and Bioassays, School of Biomedicine, FEFU, 8 Sukhanova str, Vladivostok, Primorsky region, 690950, Russian Federation; 3 Microalgal Biotechnology Laboratory, The French Associates Institute for Agriculture and Biotechnology for Drylands, The J. Blaustein Institutes for Desert Research, Ben-Gurion University of the Negev, Sede-Boqer Campus, Midreshet Ben-Gurion 8499000, Israel

**Keywords:** esophageal cancer, esophageal adenocarcinoma, squamous carcinoma, alginates, carrageenan

## Abstract

Esophageal cancer is an increasing concern due to poor prognosis, aggressive disease modalities, and a lack of efficient therapeutics. The two types of esophageal cancer: esophageal squamous cell carcinoma (ESCC) and esophageal adenocarcinoma (EAC) are responsible for an estimated 450,000 annual deaths, with over 457,000 new patients diagnosed in 2015, making it the eighth most prevalent and the 10th most fatal cancer worldwide. As esophageal cancer prevalence continues to increase, and so does the pressing need for the development of new and effective strategies for the early diagnostics, prevention, and treatment of this cancer, as well for building the innovative research tools to understand the affected molecular mechanisms.

This short review summarizes the current statistics and recent research of the problems and solutions related to the esophageal cancer, and offer a brief overview of its epidemiology, molecular alterations, and existing biomedical tools. We will discuss currently available research tools and discuss selected approaches we deem relevant to find new model systems and therapies for the future with the special focus on novel opportunities presented by the unique molecules found in algae, namely carbohydrates and lipids. Their remarkable chemical variability is connected to their striking structural and functional properties, which combined with the relative novelty of these compounds to cancer biology, warrants interest of the wide biomedical community to these molecules, especially in the esophageal cancer theory and practice.

## 1. Introduction

Esophageal cancer (EC) is the eighth most common diagnosis among all gastrointestinal malignant diseases and the sixth most common cause of cancer-related death in the world [1]. In fact, it accounts for about 4% of cancer diagnoses and for 6% of cancer deaths. The incidence of EC in the world is relatively high and on the increase year by year [2]. According to the American Cancer Society in 2017 in the United States of America there were 16,940 new esophageal cancer cases diagnosed and 15,690 deaths from EC [3].

The prognosis for esophageal carcinoma is poor, with a 5-year survival rate of 19% and only 0.9% for advanced esophageal carcinoma [4]. In males, it is the 7th most common cancer and men are up to 4 times more at risk of developing esophageal cancer than women [3]. The prevalence of esophageal cancer varies geographically and among age and race groups, with the highest risk groups being individuals over 70 years old, and black males [5]. As of 2012, more than 80% of esophageal cancer cases occurred in developing countries, and while the prevalence remains highest in Asia and Africa, occurrences in North America and Europe are on an exponential rise [6].

EC is conditionally subdivided into esophageal squamous cell carcinoma (ESCC) and esophageal adenocarcinoma (EAC). ESCC and EAC are two distinct subtypes considering geographical and demographic prevalence, etiology, as well as histopathological, epidemiologic and molecular aspects [1]. EAC arises from the metaplastic Barrett's esophagus (BE) in the context of chronic inflammation secondary to exposure to acid and bile. The main risk factors for developing ESCC are cigarette smoking and alcohol consumption. ESCC is the most prevalent type worldwide, responsible for over 80% of esophageal cancer cases [7], and particularly predominant in Asia and Africa, while EAC occurs more often in Western countries [8] meanwhile North America and Europe are on an exponential rise [6]. The main somatic genetic abnormalities showed a different genetic landscape leading to these two types of EC, thus emphasizing distinct molecular pathological mechanisms and pointing the necessity of different specific targeted therapy development. EAC is a heterogeneous cancer dominated by copy number alterations, high mutational burden and co-amplification of the receptor tyrosine kinases [4]. Genes regulating cell cycle (CDKN2A) [9], receptors to growth factors (SMAD4) [10], chromatin remodeling (ARID1A) [11] and Racl pathway (ELMO1 and DOCK2) [12] are significantly mutated in EAC, while ESCC is characterized by alterations in the mechanisms controlling terminal differentiation (KMT2D) and proliferation (FAT1 and FAT2) [13]. Meanwhile, defected TP53 and PIK3CA genes are the common feature for both EC types [12],[14].

Patients with EC may have unspecific symptoms like tiredness, nausea, weight loss, etc., at an early stage, which makes it difficult to diagnose [2]. As a result, patients often develop lymph node metastases and tumor invasion into adjacent organs at the time of diagnosis, while lack of the effective chemotherapeutic approaches available to treat ESCC patients, which additionally contribute to the poor ESCC survival [1]. Although some progress has been recently achieved in the understanding the carcinogenesis mechanisms and novel therapy approaches were proposed for the EC, there is still no effective treatment for this deadly disease and the patients' survival remains very poor [2].

## 2. Esophageal squamous cell carcinoma

ESCC is an aggressive type of epithelial cancer that is characterized by scarce overall survival and a low rate of response to the adjuvant therapy [15]. ESCC is the most frequent esophageal cancer in the world, with the highest incidence in eastern Asia and parts of Africa and poor prognosis worldwide [4]. Both the advanced disease stage at the time of diagnosis and the lack of significant molecular biomarkers to effectively stratify patients for treatment options, contribute to the poor prognosis [1].

ESCC accounts for about 90% of cases of EC worldwide and the 5-year survival rate for patients with ESCC remains generally poor despite it has improved during the past decade [1]. There were approximately 52,000 incident cases of EAC worldwide in 2012, compared to the estimate of 398,000 for the ESCC [16]. The major risk factors for ESCC are represented by tobacco smoking, alcohol consumption, genetic variations of low-activity ethanol-metabolizing enzymes (ALDH1/2), human papillomavirus infection [17], but other environmental factors also play a role in the development of this cancer, such as the consumption of hot beverages, nutritional deficiencies and limited intake of fruits and vegetables [4].

The most frequently mutated genes in ESCC were found to be TP53, TTN, MLL2, CDKN2A, PIK3CA, NOTCH1, NFE2L2, EP300, ADAM and FAM135B. These genes belong to the pathways controlling epigenetic processes (MLL2, EP300, CREBBP, TET2), cell cycle (TP53, CCND1, CDKN2A, FBXW7), as well as NOTCH (NOTCH1, NOTCH3), WNT (FAT1, YAP1, AJUBA), and receptor-tyrosine kinase phosphoinositide 3-kinase signaling pathways (PIK3CA, EGFR, ERBB2) [1].

## 3. Esophageal adenocarcinoma

EAC arises from glandular cells and represents the most lethal condition gastroenterologists face. Most EAC originates in Barrett's esophagus (BE), a pre-malignant condition. Barrett's esophagus is a metaplastic change of the esophageal mucosa from squamous to columnar mucosa with intestinal metaplasia [18]. Only 16% patients survive five-year point with the median survival time less than a year, while relatively little progress has been made in stemming the toll of this condition [16]. Adenocarcinoma was once an exceedingly rare histological type of esophageal cancer, but its incidence has increased rapidly over the recent decades in Western countries [19]. However, the reasons for this increase are incompletely understood. Many investigators have suggested that the concurrent epidemic of obesity may be at least a partially explanation for this increase [16].

EAC is most common in industrialized countries with populations of predominant European race; nearly 50% of all cases occur in Northwest Europe and North America. Incidences are highest in the United Kingdom (UK), Ireland, France, and the Netherlands, indicating a Northern European predilection. EAC is rare in Asia and Africa, but China accounts for approximately 18% of all incident cases worldwide, due to its large population. The EAC incidence has continued to increase in the West, but may be reaching a plateau. The EAC incidence—in the US was 2.5/100,000 individuals/y in 2011 [16]. The major risk factors for EAC are gastro-esophageal reflux disease and obesity, both leading to the only described precursor lesion for this cancer, namely Barrett's esophagus. Studies also showed that gastroesophageal reflux could exacerbate the impact of carcinogens, while the excess iron load and high-fat diets were also implicated in the esophageal adenocarcinoma development [20].

In addition to the classic risk factors for the gastroesophageal reflux, male gender and a number of genetic alterations were found associated with oncogenic activity. Examples include 19p13 in CRTC1, leading to the aberrant activation, as well as 9q22 in BARX1 which encodes a transcription factor which is important in esophageal specification. Furthermore, polymorphisms near TBX5 and GDF7, which encode for a bone morphogenetic protein and a transcription factor that regulates esophageal development are associated with an increased risk of BE [18].

EAC has one of the highest male-to-female ratios reported for cancers of non-reproductive organs, 7–10 to 1, significantly higher than for the major risk factors [19].

The most frequent mutational events occurred at the level of TP53 (81%), ARID1A (17%), SMAD4 (16%), CDKN2A (15%), KCNQ3 (12%), CCDC 102B (9%) and CYP7B1 (7%). Importantly, large-scale genetic events are frequently observed in EACs are chromothripsis (30%), kataegis (31%), and complex rearrangement events (32%) [4].

## 4. Towards understanding the molecular mechanisms of EC

Analysis of the molecular mechanisms specific to EC and their functional consequences common to various cases falling into this type of carcinogenesis, is essential to move further in understanding of these diseases, as well as to develop optimal strategies for diagnostics and treatment. Since it is virtually impossible to cite all relevant literature, we will focus on the few recent studies unraveling different aspects of the esophageal cancer molecular biology, which could facilitate breakthroughs in future medicine.

The importance of molecular profiling of cancer types is emphasized by the possibility of developmental risk prediction by investigating several single nucleotide polymorphisms (SNPs) characterizing a particular cancer type. Specifically, Arg72Pro substitution in p53 gene disrupts apoptosis and is associated with elevated risk of EAC development and reduced response to chemotherapy [21]. T309G substitution in the promoter region of MDM2 gene, regulating the p53 destruction, results in the enhanced MDM2 transcription, which causing the reduced apoptosis in response to the DNA damage [22]. Additionally, mutations in several Fanconi anemia-predisposing genes, such as heterozygous indels in FANCD2 (p.Val1233-del), FANCE (p.Val311SerfsX2) and FANCL (p.Thr367AsnfsX13) were shown to correlate with enhanced ESCC risk [23]. In tobacco smokers, special genomic variants of xenobiotic metabolizing enzymes—cytochrome P450 (CYP3A5) and sulfotransferase (SULT1A1*2/*2), specifically relate to increased ESCC risk, emphasizing the importance of combinatorial influence of genetic predisposition and environmental factors for cancer development [24]. The same way bile acid exerts its dismal effect, so reflux present in patients with BE may cause cell transformation by activating PIPLCγ2, MAPK kinase, and NADPH oxidase NOX5-S, thus causing DNA damage and gene mutation contributing to the development of EAC.

Recent work [25] identified both TGM3, coding for transglutaminase 3 involved in differentiation of the stratified squamous epithelia, and HSPB1, encoding for the small heat shock protein protecting cells from apoptosis, as expressed at higher levels in squamous epithelium compared to EAC. In contrast, another molecular chaperone—AGR2 (Anterior gradient 2) was found to be expressed in gastric epithelium and EAC, with no expression observed in squamous epithelia. Similarly, a protein chaperone HSPA5 involved in correct protein folding was found to be expressed in both EAC and gastric epithelium. Indeed, AGR2 has functions in protein homeostasis and secretion [25]. A member of Rho protein family, ARHGDIB, was found overexpressed in EAC sections compared to normal squamous and gastric tissues. The cancer antigen EpCAM was highly expressed in EAC cells compared to surrounding normal tissues. EpCAM was also found to be highly specifically expressed in lymph node metastases compared with surrounding normal lymph nodes raising the possibility that it could be exploited to enhance clinical grading using novel techniques [25].

In addition to genetic events, a wide range of epigenetic regulators, including miRNAs, were shown to contribute significantly to cancer phenotypes. For instance, it was established that extracellular vesicles (EV)-delivered miRNAs can promote tumor progression and metastasis. Specifically, miR-21, miR-25, miR-93, miR-192 and miR-210 are well-known oncogenic miRs, which have also been studied as biomarkers for several types of human cancer. These effects were mediated by EV miR-25 and miR-210. Therefore it was suggested [26] that EVs may serve as promising cancer biomarkers and potential therapeutic tools, and that miR-25 and miR-210 constitute potential molecular targets in esophageal and gastric cancers diagnosis and treatment.

Studies in animal models of gastrointestinal cancer [27] have demonstrated that CCK2R signaling can accelerate tumorigenesis *in vivo*, such as in gastrin-overexpressing INS-GAS mice that develop proximal gastric cancers. Hypergastrinemia could stimulate CCK2R+ cells in BE tissue to proliferate, and increased proliferation correlates with less differentiation, less mucus cell metaplasia in BE areas, ultimately leading to accelerated malignant transformation. These findings suggest that elevated serum gastrin levels in BE patients warrant further studies. Gastrin stimulation appears to have the same proliferative effect on Barrett's epithelium, however here this phenomenon was found to be longer lasting. Hypergastrinemia promotes progression and dysplasia in Barrett's-like esophagus in a mouse model. Mouse models limitations warrant caution in extrapolating these data to human BE. However, these findings suggest that in patients with high gastrin levels in BE further longitudinal studies should be performed, as well either trials of CCK2R inhibitors or more selective use of PPIs should be considered [27].

## 5. Towards better treatment of esophageal cancer

Traditional methods of malignant neoplasms treatment are radiation therapy, chemotherapy and surgical intervention, as well as their combinations. These methods are also used for the esophageal cancer treatment, but the effectiveness of such treatment depends on many factors, including the stage of the tumor and its location, as well as the age of the patients, their health state and life choices.

Surgical methods involve partial removal of a part of the affected esophagus. Extirpation procedure consists of the esophagus removal along with the affected lymph nodes and a number of adjacent tissues; however this operation is possible only in 5% of patients with cancer due to the late diagnosis, advanced age, and the presence of other serious diseases [28]. In addition to surgical removal, less extensive surgery is performed to remove small tumor foci via endoscopy, widening of the narrowed part or recanalization of a partially or completely tumor-blocked esophagus [29]. The 5% patients benefit from radiotherapy resulting in the prolongation of their survival rate to around 5-years mark. In other cases, radiotherapy substitutes the surgical intervention when the latter is impossible and additionally, as a means of palliative treatment. In the cases when it is combined with the surgical treatment methods, special radiological regimens should be designed before and after surgery. Radiotherapy is used before the operation in case of undifferentiated or infiltrative forms of cancer, as well as for the tumors localized in the middle third part of the esophagus, which is the anatomical region that complicates the surgical approach to radical tumor tissue removal. After the operation, radiotherapy is indispensable in the cases when the radical surgical tumor removal is not feasible or if there is a risk of leaving residual cancer cells, e.g. when the border between cancer and healthy tissue is not apparent [28]. Chemotherapy alone does not provide significant results and therefore it is used in conjunction with other treatments. For example, chemotherapy drugs in conjunction with radiation therapy lead to the complete regression of the tumor in 20% of cases. Also in some disease cases, surgery can be performed after the chemoradiotherapy [30].

The esophageal cancer is considered as one of the biggest challenges for surgical approach regarding the complexity of applying the treatment precisely to the tumor localization, leading to successful results solely in cases of small tumors, with no metastases. However, operations that result in tumor removal, but leaving behing spikes, tumor emboli, and/or metastases, are considered palliative. Thus, the treatment is effective only at the early stages of tumor development, which is difficult to achieve, considering the current state of progress in diagnosis for these cancers. The symptomatology characteristic of esophageal cancer manifests only when a significant tumor spread leads to esophageal dysfunction, thus almost completely precluding the timely treatment start. The failure of the standard therapies repertoire to achieve high survival rates has led to a paradigm shift towards targeted therapy disrupting a particular tumorigenesis mechanism, leading to the emerging interest to small molecules, e.g. inhibiting different receptor tyrosine kinases or antibodies against growth factor receptors. The most prominent examples are cetuximab [31] and panitumumab targeting EGFR [32], trastuzumab binding to HER2 [33], bevacizumab inhibiting tumor neovascularization by blocking VEGF [34], however, thus far only trastuzumab was approved for the esophageal cancer treatment [35]. Glivec, gefitinib and erlotinib are the examples of therapy utilizing tyrosine kinase inhibition, but none of them showed promising enough results to warrant their further development [36]–[38]. Although a lot of effort was put into the development of the targeted therapies for the esophageal cancer, the remarkable breakthrough in terms of overall survival rates was achieved for neither ECSS nor EAC patients, suggesting the plasticity of cancer survival mechanisms allowing these diseases to evade the inhibition. This presses for necessity to search for substances exerting complex mechanisms of action via disrupting several molecular pathways concurrently.

The area of BE therapeutic intervention is rapidly evolving. Endoscopic eradication therapies have been already proven to be effective in patients with BE/EAC, while new therapies are arriving. BE containing HGD and/or early-stage EAC can be treated endoscopically to replace the surgical esophagectomy. Moreover, recent treatment strategies, including a de-escalation strategy for lower-risk patients and escalation with a follow-up for higher-risk patients, have been established. The main objective of endoscopic therapy should be the elimination of all intestinal metaplasia because the recurrence of neoplasia appears to be higher in patients who didn't undergo full BE eradication [39].

In summary, the abovementioned methods of treatment are traumatic for the patient because of their invasive and toxic nature, while they do not significantly prolong the patient's life. Therefore, it is logical to search for other methods, in particular relying on hypothetical chemical compounds that affect only cancer cells and do not affect normal healthy cells. Compounds of natural origin are especially attractive in this light, especially in the cases of smaller toxicity for the patient's body, synergistic action of the molecules found in the natural extracts and their semi-purified fractions and in some cases complementary structural components, allowing for their convenient delivery and prolonged exposure at the affected sites, e.g. via orally administered tinctures and suspensions known from traditional medicine.

A prominent example of such substances is the alginic acid which is a naturally occurring polysaccharide derived from brown algae. This biopolymer has a number of biologically active properties. For instance, it is not digested by the stomach to be transited through the intestine, neutralize hydrochloric acid and exert its' antimicrobial effect. Moreover, alginic acid and its salts are used as food additives because of their thickener properties. Thanks to these properties, this compound is included in a wide variety of medicines, such as antacids, successfully used to treat the gastroesophageal reflux which often is a cause of Barrett's esophagus (BE) development, a pre-malignant condition of the most cases of EAC [18],[40].

In addition to alginic acid, there is another promising compound derived from algae referred to as carrageenan, which is a sulfated polysaccharide. Like alginic acid, carrageenan is widely used as a food additive. Furthermore this substance has a wide spectrum of actions, for example, immunomodulating and anti-inflammatory activities. Additionally, it lacks toxic effects on the human body, possesses biocompatibility, and relatively easily extracted [41].

While the optimal objective of selective therapy is to entirely kill cancer cells without causing significant harm to healthy tissue, non-cytotoxic options may also be beneficial [42]. Due to the above described properties, algal polysaccharides are one of the most promising natural compounds with respect to the potential effects on the esophageal cancer, since it is able to locally regulate the immune response of the organism at the tumor site, and also to protect esophageal tissues from inflammation and thereby facilitating the disease regression. Anyhow further identification of new biomarkers and therapeutic targets is essential to optimize the current therapeutic regimens for treating such this spectrum of deadly diseases tools [2].

## 6. Polysaccharides and fatty acids from algae as a source of the innovative solutions for the esophageal cancer treatment

Recently the research and development of new anti-cancer drugs have accelerated, and natural chemo-diversity is regaining the momentum as a valuable resource for the drug discovery. At present, about 60% of the commercially available anticancer drugs are of natural origin [43]. Anti-proliferative drugs derived from natural compounds, including doxorubicin, bleomycin, daunomicin, vincristine, mytomicin C, vinblastine, as well as many others, play an important role in curative cancer chemotherapy for a number of solid tumors and hematological malignancies [31],[44]. Nowadays many unique chemical compounds of marine origin were reported to possess various biological activities. Some of them were already approved as anticancer drugs and others are under pre-clinical and clinical trials that promise to lead to the development of the new high quality pharmaceuticals [45].

Different groups of bioactive molecules with antitumor activity have been isolated from algae, including fatty acids, polysaccharides, phenolic compounds, carotenoids and terpenoids. These compounds have showed anti-proliferative activity in human cancer cell lines *in vitro*, as well as inhibitory activity in tumors in animal models [46]–[50]. Polysaccharides comprise the main component of the algae biomass and serve various biological functions. The most valuable biologically active polysaccharides of brown algae are alginic acids, laminarans and fucoidans. In recent years, fucoidans have been the subject of intensive research due to their low toxicity and diverse biological activities that can be used to develop innovative medicines [51].

In addition, some marine algae species are regarded not only as food but also often are used to treat stomach disorders [47]. For example, biologically active polysaccharides from Saccharina japonica, such as fucoidans, laminarans and alginic acids, have attracted attention recently due to their remarkable biological properties including immunoregulation, hypolipidemic effects, as well as antioxidant, antibacterial, anticoagulant and antiviral activities. Other marine algae can be used as sources of biologically active substances and new drugs as well. So, the antitumor potential of twelve algae extracts from Portuguese coast was studied in an *in vitro* model of human hepatocellular carcinoma (HepG-2 cells), and few of them showed significant anti-proliferative action on HepG-2 cells [52].

It was shown that fucoidan from brown alga Sargassum duplicatum is effective against the colon cancer cells colony formation *in vitro* [51]. Other study demonstrated that water-soluble fraction of polysaccharides isolated from the alga Capsosiphon fulvescens exhibits antioxidant and anti-tumor activities, inhibits cell proliferation, and induces apoptosis of gastric cancer cells by modulating the IGF-IR signaling and the PI3K/Akt pathway [47]. A cancer chemopreventive activity of the polysaccharide extracts of brown alga Sargassum asperifolium was also revealed [53].

Additionally fucoidans from various brown algae species effectively inhibit the proliferation of human colon cancer cells (HT-29, HCT 116, HCT 15) [54],[55] and human breast cancer cells (MCF-7 and MDA-MB-231) [54],[56]. Commercial fucoidan from Cladosiphon novae inhibits the growth of breast cancer cells (MCF-7, MDA-MB-231), cervical cancer (HeLa) and fibrosarcoma (HT1080) [57]. Furthermore, brown alga fucoidans possess antimetastatic activity significantly reducing the adhesion of human breast cancer cells MDA-MB-231 [58].

It has been shown that molecular mechanism behind the antitumor effect of algal polysaccharides is linked to their ability to induce apoptosis in tumor cells [59],[60]. For instance, when applied to human adenocarcinoma cells, fucoidan induces apoptosis accompanied by the inhibition of Bcl-2 and Bcl-xL expression, loss of mitochondrial membrane potential, caspases activation and PARP degradation. In addition, morphological changes characteristic of autophagy and the formation of autophagosomes were detected in cells treated with fucoidan. The material to be destroyed is isolated and delivered to their processing site, namely lysosomes [59],[60].

It has also been shown that fucoidan effectively inhibits the proliferation of human intestinal cancer cells HCT 15 and induces the apoptosis. After treatment with fucoidan, apoptogenic processes occur: DNA fragmentation, chromatin condensation, and an increase in the population of sub1-G1 diploid cells. The level of expression of anti-apoptogenic Bcl-2 protein is reduced by the action of fucoidan, and at the same time proapoptogenic protein Bax levels are increased. In addition, activation of the initiator caspase 9 and the subsequent activation of the effector caspase 3 (an apoptotic agent) were registered following the fucoidan action. Caspase 3, in turn, inactivates the enzymes involved in DNA repair, via cleaving PARP, and thereby is inducing human intestinal tumor cells apoptosis. It was shown that under the action of fucoidan, the apoptosis induction was associated with the phosphorylation of ERK 1/2 and p38 protein kinases, as well the PI3/AKT signaling cascade inactivation [59].

Marine red algae are a source of biologically active sulfated polysaccharides—carrageenans which share bioactivity with fucoidans isolated from brown algae, in particular, anticancer activity *in vitro* and *in vivo* against several types of tumors [41],[61]–[64].

The peculiarities of the carrageenan molecular structure allow it to interact with receptors on the surface of the immune cell and to influence the regulation of cellular and humoral immunity [41]. Carrageenans are known to stimulate the immune mediators' biosynthesis, including different cytokines, either pro-inflammatory or anti-inflammatory depending on their structural type [65]. For instance, we have previously revealed the ability of κ/β-carrageenan to stimulate the induction of anti-inflammatory cytokine IL-10 in human and mouse blood cells ex vivo and *in vivo* [66]. Carrageenan inhibits the pro-inflammatory responses of the body: Due to the influence of its sulfated groups on the complement system, inflammation can be mitigated [67]. Moreover, carrageenans prevent leukocytes migration [68]. Carrageenans have a dose-dependent effect: At higher concentrations, these substances have no immunosuppressive effect [67], while stimulation and maturation of suppressor macrophages occur at lower concentrations. Carrageenans are known to influence local immunity, including the effects on Toll-like receptors [69] of macrophages and neutrophils that interact with bacterial endotoxins in the gastrointestinal tract. The hyperactivation of these receptors leads to an uncontrolled immune response. Carrageenan molecules also prevent the activation of these receptors and suppress the hyperactive immune response thereby protecting the body exposed to endotoxins.

Certain algal polysaccharides, such as alginates and carrageenans are widely utilized in medical industry as excipients for making drug tablets due to their gel-forming properties [70]. Carrageenans are used to reduce the amount of polymorphic transformation in tableting [71], to control release and delivery [72] and to achieve interactions with other drugs to achieve systems with modified release [73]. In addition, complexation with these polysaccharides promotes the protection of medical agents from digestion in the gastrointestinal tract and provides their prolonged action [70],[74],[75]. Furthermore, these polysaccharides are characterized by a number of useful properties of their own, including the ability to stimulate tissue regeneration and also anti-inflammatory, gastroprotective, and anti-ulcerogenic properties that in some cases can provide increased therapeutic effects of medications. Regarding this, for example, the pronounced gastroprotective effect of soluble polyelectrolyte carrageenan-chitosan complexes was shown using the model of stomach ulcers induced by indomethacin in rats [76]. Such properties of natural high molecular weight polyionic polysaccharides are likely related to their ability to associate with the protective layer on the surface of the mucous stomach membrane, protecting it from the direct contact with the ulcerogenic agent.

In addition, it should be noted that algal polysaccharides, such as fucoidans, alginates and carrageenans belong to soluble food fibers, which play a significant role in homeostasis regulation and gastrointestinal diseases prevention, as well as metabolic and functional disorders [75],[77]. Dietary fibers are widely known to have high absorption ability and antioxidant activity. These natural products promote excretion of endo- and exotoxins from the organism, bind and remove cholesterol, bile acids, heavy metals and carcinogenic substances, reducing toxins interaction with the intestinal mucosa, lowering the severity of the intoxication syndrome and inflammatory dystrophic changes of mucosa [78],[79]. The ability to normalize the function of gastrointestinal tract and reduce the level of free ammonia, is characteristic for other carcinogens, formed during food fermentation, allows the dietary fibers formation to prevent the development of colon cancer and other intestinal cancers, esophageal cancer, intestinal diverticulosis, esophageal hernia and other intestinal diseases [77],[78]. Furthermore, algal polysaccharides are characterized by the ability to react with acidic stomach contents and form a gel with pH closed to neutral. The gel creates a protective barrier on the surface of the stomach contents, preventing the occurrence of gastroesophageal reflux and ulcers. So, entering the esophagus, outstripping the rest of the stomach contents, it reduces the irritation of the esophagus mucosa [80].

Inflammation is now recognized to be a critical component for tumor progression and one of the recently added “hallmarks of cancer”. Epidemiological and genetic studies support the link between chronic inflammation and tumor progression [81]. People with chronic inflammatory diseases are at increased risk of developing cancer of the respective inflamed tissue indicating that inflammation is, at least in part, the cause and not an effect of cancer development [82],[83]. For example, chronic inflammation of the colon (ulcerative colitis) markedly increases the risk of developing colon cancer later in life [84],[85]. Conversely, anti-inflammatory drugs decrease the risk of developing certain cancers. For instance, non-steroidal anti-inflammatory drugs (NSAIDs) reduce the risk of developing colon, breast, lung and prostate cancer by reducing tumor associated-inflammation [79].

Earlier we have shown protective and anti-inflammatory effects of gelling polysaccharide from red alga Chondrus armatus following the oral administration to treat acid-induced colitis in mice [86]. In addition, distinct types of carrageenans from red algae from Gigartinaceae and Tichocarpaceae families possess the *in vitro* scavenging effects in relation to hydroxyl radicals, superoxide anion, nitric oxide and hydrogen peroxide [87].

A link between development of esophageal cancer and chronic esophagus inflammation was previously established [88]. Inflammation may contribute to cancer development through multiple mechanisms, including DNA damage, angiogenesis, and promotion of cellular proliferation and inhibition of the apoptosis. Inflammatory processes also lead to generation of reactive oxygen species (ROS) which may cause inactivating mutations in tumor suppressor genes or post-translational modifications in DNA repair proteins, thus promoting carcinogenesis. Inflammatory conditions of the esophagus, namely reflux esophagitis and BE, are implicated in the development of EAC. The inflammatory link with EAC is further strengthened by the observation that regular use of NSAIDs is associated with decreased risk of this disease [89]. Interesting observations between connection of diet type and risk of esophageal cancer were published [90],[91]. Such observations might additional indicate significant role of inflammation in development of esophageal cancer, since Mediterian, low meat—hi fish diet is also known as low-inflammatory diet, due to high content of omega-3 fatty acids in fish oil.

Algae as original as carriers of many types of biologically active molecules and producer of omega-3 fatty acid that translated by food chain and accumulated in form of fish oil might be considered as highly promising therapy for prevention and treatment of various cancer types, including esophageal cancer.

In summary, cancer development depends on the combinatorial influence of genetic predisposition and environmental factors, as well as the effects of various concomitant diseases [24]. In this regard, the treatment of esophageal cancers requires a comprehensive approach. Algal polysaccharides, being completely non-toxic, possess a variety of useful properties, increase the overall resistance and protective functions of the organism, positively affect the gastrointestinal tract and digestive system, protect the esophagus mucosa, stomach and intestines from the effects of various toxins, regulate pH and content bile acid. Furthermore algal polysaccharides exhibit antitumor action, can influence on apoptosis, proliferation and possess antimetastatic activity. Thus, the abovementioned unique combination of properties prove that these substances can be considered as potential drugs for the treatment and prevention of complex-action esophageal cancers, since they can be used both as preventive drugs and as medications in the early stages of the disease or previous pathological conditions.

## 7. Conclusions

Esophageal cancers represent a substantial health problem in the world due to their increasing incidence and poor prognosis. Nowadays there are not enough cell based systems to model esophageal cancers in the *in vitro* functional studies. Even making a standard flat patient-derived cancer cell tissue culture in still presents a significant challenge for this cancer type. However it is essential to develop new *in vitro* model systems that mimick natural tumor conditions *in vivo*, such as local intra-tumor hypoxia, immune compound creating cancer protective immunosuppressive milieu and extracellular matrix forming an appropriate growth niche for the tumor cells. Prominent examples of these approaches for the other cancer types include tissue culturing in aggregates and hydrogel scaffolds allowing for modeling tumor growth to resemble primary conditions *in vivo*. Specifically, hydrogels scaffolds are capable of adjusting their mechanical, compositional and structural properties, thus enabling more accurate representation of the native tissues. Hydrogels containing extracellular matrix compounds close to native, such as pectines and collagens, have proven effective in regulating cell behavior and providing innovative tools for controlled differentiation of various cell types, which are not possible with conventional 2D cultures [92]. Meanwhile, culturing in aggregates allows for creating tumor microenvironment arranged by immunocompetent cells and stromal cells thus providing means for modelling characteristic intra-tumoral hypoxia and interaction between immune and stromal cells within the tumor [93],[94].

Screening natural extracts is very promising as it holds the promise of enhancing effectiveness of chemotherapeutic drugs by increasing immune responsiveness or cancer cell susceptibility. Algal polysaccharides are remarkably promising substances for the development of the treatment strategies for the cancers of the esophagus, since they are able to locally regulate the immune response of the organism to the tumor and at the same to protect esophageal tissues from the inflammation known to exacerbate the disease progression. We therefore expect significant progress as a result of creating new cell-based models and screening of the potential antitumor agents in these models in order to shed light on the mechanisms of carcinogenesis and possibly find promising strategies to cure these deadly cancers. Moreover, we expect that natural products derived from algae will score highly in such screenings.
